# The First Whole Genome Sequencing of Historical Lichen Specimens Enables Genome‐Wide Analysis of Fungal and Algal Symbionts

**DOI:** 10.1002/ece3.72216

**Published:** 2025-09-22

**Authors:** Mieko Kono, Yoshihito Ohmura, Yohey Terai

**Affiliations:** ^1^ Research Center for Integrative Evolutionary Science SOKENDAI (The Graduate University for Advanced Studies) Hayama Kanagawa Japan; ^2^ Department of Botany National Museum of Nature and Science Tsukuba Ibaraki Japan

**Keywords:** genome sequencing, historical specimens, lichen, type specimens

## Abstract

With the advent of high‐throughput sequencing techniques, natural history museums and herbaria have become new frontiers for genetic research. Genomic information from historical specimens has provided evidence to solve significant questions in broad research areas. However, access to such valuable genetic resources remains limited in lichens due to experimental challenges in extracting and amplifying highly degraded DNA in historical specimens. So far, only a handful of studies have reported successful sequencing of several short genetic markers from historical lichen specimens despite the increasing importance of genetic information in lichenology. Here, we aimed to establish an efficient method for sequencing the whole genome of historical lichen specimens. We modified a method used in ancient DNA studies and sequenced the whole genome of *Usnea* and *Cladonia* specimens, including lectotype and holotype. Our approach shows that 2.7%–23.3% and 3.0%–11.8% of the total sequenced reads originate from the genomes of fungal (mycobiont) and algal (photobiont) symbionts, respectively. The mycobiont‐ and photobiont‐derived reads are comprised of DNA fragments shorter than 46 bp, covering 73%–99% and 92%–99% of the mycobiont and photobiont reference genomes, respectively. We retrieved 792,245 and 410,705 Single Nucleotide Variant sites (SNVs) to perform phylogenetic analysis of the *U. hakonensis* and *C. kurogawae* mycobionts, respectively. We also demonstrated experimental modifications that improved proportions of symbiont‐derived reads within sequenced data. We believe that our method is applicable to lichen specimens in a broad range of ages and taxonomic groups, thereby potentially converting historical lichen specimens into resources of genome‐wide studies.

## Graphical abstract

We established an efficient method for sequencing the whole genome of historical lichen specimens. By using this method, we successfully retrieved genomic data to perform phylogenetic analysis of both mycobiont and photobiont.

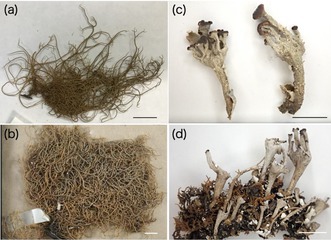

## 1 Introduction

Biological specimens in natural history museums and herbaria have contributed greatly to improving our understanding of the natural world. They have been the resource of biological information that helps to address key scientific questions, often many years after they were collected. Furthermore, recent advances in high‐throughput sequencing technologies have unlocked the potential of historical biological specimens by enabling the access to unprecedented amounts of genomic information. Historical DNA, ranging from individual genes to the entire genome, has provided significant evidence not only to traditional taxonomic and phylogenetic studies (Prosser et al. 2016) but also to evolutionary (Shpak et al. 2023), population genetics (Ghimire et al. 2024; Stuart et al. 2022; Bendiksby et al. 2014), conservation, and biodiversity studies (Card et al. 2021; Raxworthy and Smith 2021). However, despite the potential versatility of historical DNA in broad research areas, its implementation is still limited to particular groups of organisms due to experimental challenges in extracting and sequencing DNA that progressively degrades over time (Dabney et al. 2013; Mullin et al. 2023; Heintzman et al. 2014). Mullin et al. (2023) reported that in museum entomological specimens, DNA undergoes a rapid and large‐scale post‐mortem reduction in fragment size, followed by reduction at a much more gradual time‐dependent rate. The DNA degradation seems to progress at different speeds among different specimen types, as Weiß et al. (2016) have estimated a per nucleotide per year decay rate of 1.66 × 10^−4^ in plants, which is six times faster than the rate estimated from ancient bones, 2.71 × 10^−5^ (Allentoft et al. 2012). These differences in DNA quality among specimens indicate the importance of adjusting DNA extraction and sequencing protocols according to specimen types.

In lichens, Kistenich et al. (2019) noticed that DNA fragments are mostly shorter than 50 bp in specimens older than 50 years. This size is comparable to DNA extracted from more than a hundred‐year‐old insect or fungal specimens (Shpak et al. 2023; Mullin et al. 2023; Shumskaya et al. 2023). In addition, previous studies pointed out that lichens may contain specific substances that interfere with DNA extraction and/or PCR amplification (Kistenich et al. 2019; Cubero et al. 1999; Sohrabi et al. 2010). Due to these experimental difficulties, only a handful of studies have successfully sequenced several short genetic markers (Bendiksby et al. 2014; Kistenich et al. 2019; Sohrabi et al. 2010; Gueidan et al. 2019; Gueidan and Li 2022), and so far, there has been no report of whole genome sequencing of historical lichen specimens. DNA sequences have been particularly useful in the identification of lichens with morphological and chemical characters that often present high infraspecific plasticity and interspecific convergence (Del‐Prado et al. 2019; Kelly et al. 2011). Indeed, studies using multi‐locus or genome‐wide sequences have unveiled cryptic diversity in some lichen groups (Kanz et al. 2015; Zhao et al. 2017). Therefore, establishing methods to obtain genomic information from well‐curated museum lichen specimens will not only provide a powerful tool for species identification but also provide resources crucial for future lichenology studies.

In this study, we aimed to establish an efficient method for obtaining whole genome data from historical lichen specimens. For this purpose, we chose two specimens of *Usnea hakonensis* Asahina collected in 1952 and that have been stored in the National Museum of Nature and Science in Japan for the following reasons. (1) In resequencing of fragmented DNA, it is vital to have a reference genome for the alignment. In our previous studies, we have determined the whole genomes of both the mycobiont and the photobiont of *U. hakonensis* (Kono et al. 2017, 2020). (2) Identification of *U. hakonensis* through morphological and chemical keys and short genetic markers has been controversial due to variation among collected individuals (Ohmura 2001, 2012). Therefore, genome‐wide data of well‐curated museum specimens will help to address future challenges in species delimitation. We established experimental protocols based on a method of ancient DNA optimized for recovering heavily degraded DNA (Xiaokaiti et al. 2024). We further sequenced specimens of *Cladonia kurokawae* Ahti & S. Stenroos and *C. subconistea* Asahina to test the versatility of the protocols. Successful sequencing of the lichen species from two different families (Parmeliaceae and Cladoniaceae) indicates future applications of this method to diverse and precious museum lichen specimens.

## 2 Materials and Methods

### 2.1 Sample Information

**FIGURE 1:**
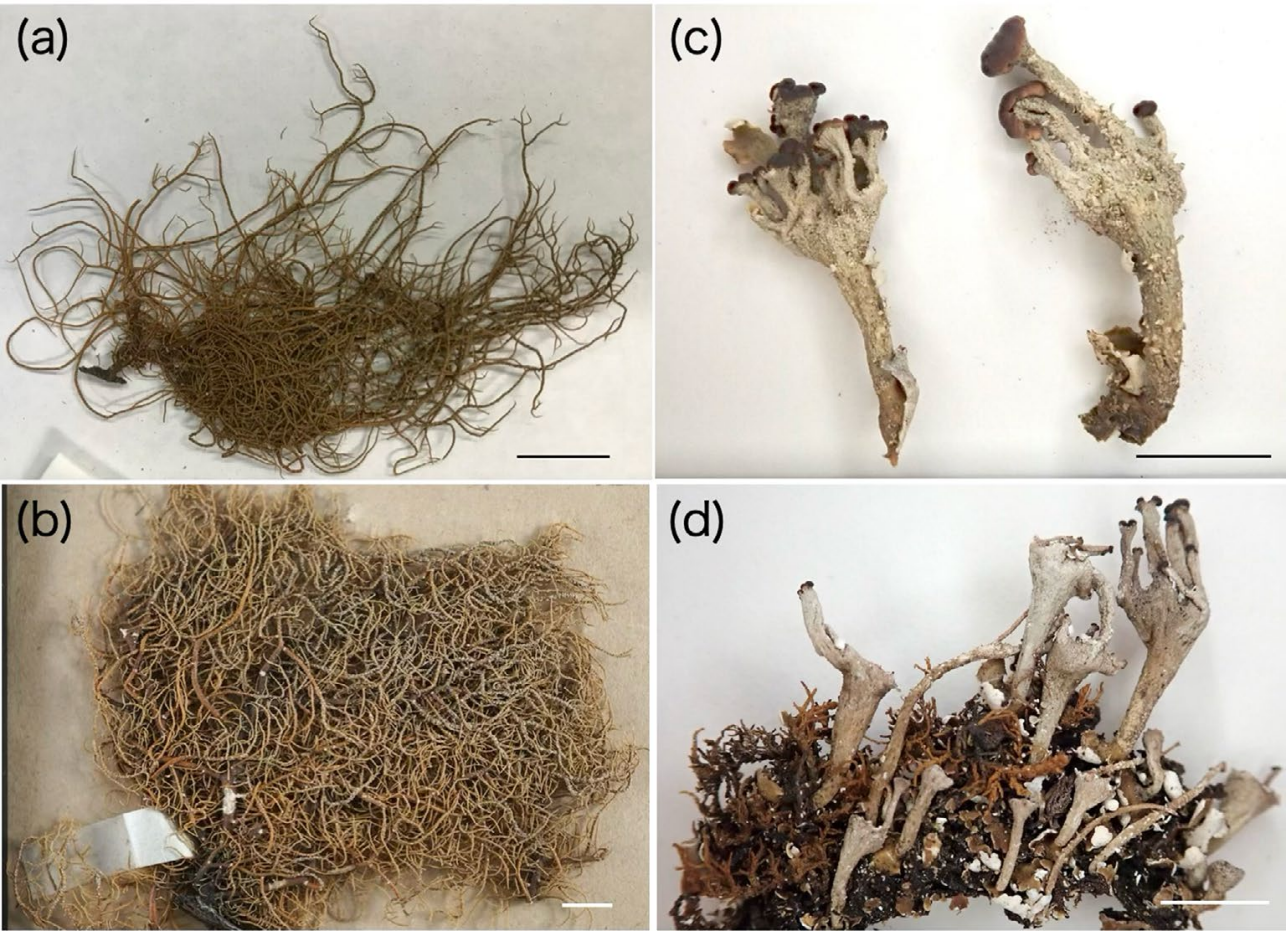
Specimens used in this study. (a) UhA (TNS‐L 21776A) (b) UhiL (TNS‐L 119447, lectotype) (c) CkuR (TNS‐L 27578, holotype) (d) Csu1 (TNS‐L 27557, lectotype). Scale bars are 0.5 cm.

Two specimens of *Usnea hakonensis* Asahina, *Cladonia kurokawae* Ahti & S. Stenroos, and *C. subconistea* Asahina, hereafter referred to as UhA (TNS‐L 21776A) and UhiL (TNS‐L 119447, lectotype), CkuR (TNS‐L 27578, holotype), and Csu1 (TNS‐L 27557, lectotype) were used in this study (Figure 1). Both *U. hakonensis* specimens were collected in the same location (Hakone, Kanagawa, Japan) on the 15th of July 1952. The CkuR was collected in Nakasato, Ibaraki, Japan on the 15th of May 1950, and Csu1 was collected in Mt. Yatsugatake, Nagano, Japan on the 28th of May 1926. The specimens have been stored at 22°C and 40% humidity in the herbarium of the National Museum of Nature and Science (TNS), Tsukuba, Japan. The herbarium had been fumigated once a year with methyl bromide, later replaced by sulfuryl fluoride.

### 2.2 Whole Genome Sequencing of *Cladonia kurokawae*

We determined the whole genomes of *C. kurokawae* mycobiont and photobiont as reference genomes for the *Cladonia* specimens. Independently cultured mycobiont and photobiont colonies were disrupted with stainless steel beads (SUB‐30, TOMY) and TOMY Micro Smash MS‐100 (TOMY). DNA was extracted using the DNeasy Plant Mini kit (QIAGEN) following the manufacturer's instructions. DNA libraries were constructed using the NEBNext Ultra II DNA Library Prep Kit for Illumina and the NEBNext Multiplex Oligos for Illumina (New England Bio Labs) following the manufacturer's instructions. The libraries were sequenced on an Illumina Novaseq X platform with 150 bp paired‐end reads. Genomes were *de novo* assembled using CLC Genomic Workbench v23.0.2 (QIAGEN) with a change in word size to 64 from the default settings. Completeness of the genomes was assessed using gVolante version 2.0.0 (Nishimura et al. 2019, 2017).

### 2.3 Optimization of DNA Extraction Protocols

#### 2.3.1 DNA Extraction From Usnea Specimens

Amounts of the specimens used in DNA extraction are listed in Table 1. Branches of *Usnea* specimens were briefly cut and collected into 1.5 mL tubes using sterilized scissors and tweezers. The samples were then disrupted at 4000 rpm for 30 s using stainless steel beads (SUB‐30, TOMY) and the TOMY Micro Smash MS‐100 (TOMY). Proteinase K and Buffer ATL from the DNeasy Blood & Tissue Kit (QIAGEN) were mixed according to the manufacturer's instructions, and the mixed Proteinase K‐Buffer ATL working solution (lysis buffer) was added to the disrupted samples. The tubes were incubated at 37°C for 24 h. After the incubation, the lysate was centrifuged at 5000 rpm for one minute. The supernatants (DNA extracts) were collected into 1.5 mL DNA low binding tubes (DNA LoBind Tubes, Eppendorf) and were subjected to DNA purification.

**TABLE 1:** The experimental setups of DNA extraction and library construction.

Specimen	Library name	DNA extract name	Input material (mg)	DNA concentration (ng/μL)^a^	Input DNA^b^ (ng)	PCR cycles^c^	Library concentration (ng/μL)
UhA	UhA1	supUhA1	4	0.384	6.91	10	2.19
UhA	UhA2	supUhA2	16	0.248	2.23	11	9.27
UhA	UhA3	supUhA2	16	0.248	2.23	11	9.33
UhA	UhA5	supUhA5	34	3.18	6.36	10	3.40
UhA	UhA24	supUhA24	34	0.644	6.44	10	2.66
UhiL	UhiL1	supUhiL1	17	1.57	10.99	10	6.96
UhiL	UhiL2	supUhiL1	17	1.57	7.22	10	9.95
UhiL	UhiL3	supUhiL1	17	1.57	7.54	10	11.12
CkuR	CkuR1	supCkuR	19	6.16	12.32	8	8.97
CkuR	CkuR2	supCkuR	19	6.16	12.32	7	2.86
CkuR	CkuR3	supCkuR	19	6.16	12.32	7	3.84
Csu1	Csu1_1	supCsu1	8	1.74	6.96	8	4.18
Csu1	Csu1_2	supCsu1	8	1.74	6.96	7	2.20
Csu1	Csu1_3	supCsu1	8	1.74	6.96	7	2.11

^a^ Concentration after the DNA purification.

^b^ Amount of DNA used in the library construction.

^c^ PCR cycles run for the library amplification.

#### 2.3.2 Maximizing the Yield of Target DNA

**FIGURE 2:**
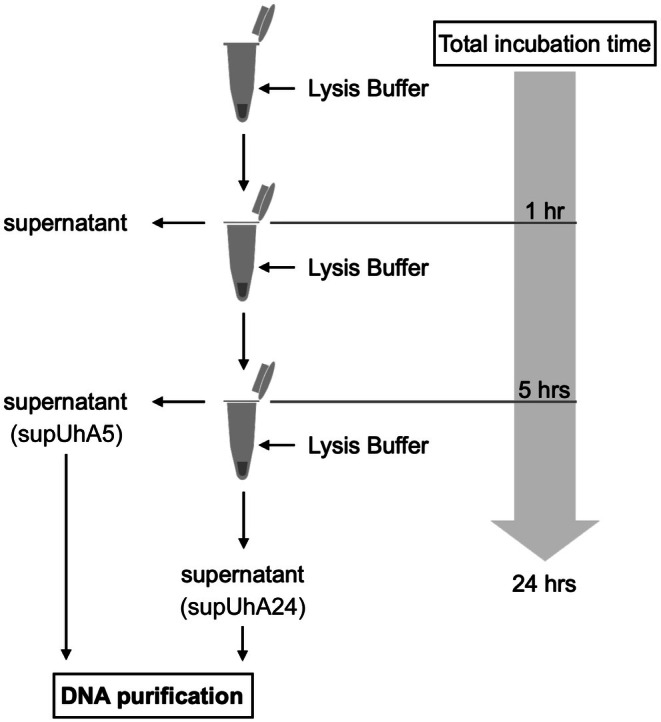
We modified the DNA extraction protocols in order to optimize the yield of symbiont‐derived reads. After an hour of incubation, the first supernatant was removed as an aliquot likely to contain a large proportion of non‐target DNA. The proportions of the target DNA in the second and third supernatants collected after five and 24 h of incubation were examined by iSeq sequencing.

To increase the proportion of DNA from the mycobiont and photobiont, we modified the lysis step of the above DNA extraction protocols based on a method of ancient DNA extraction (Xiaokaiti et al. 2024) that removes DNA from non‐target organisms (mostly bacteria) on the surface of samples by changing lysis buffer several times during the lysis step. To examine the effect of buffer exchange, we collected the supernatant of lysates after one, five, and 24 h during the incubation at 37°C (Figure 2). At each time point, the lysate was centrifuged at 14,000 rpm for 2 min. The supernatant was collected into a new 1.5 mL low‐binding tube, and the pellet was resuspended in fresh lysis buffer. The collected supernatants (DNA extracts: supUhA5 and supUhA24 in Table 1) were subjected to DNA purification.

#### 2.3.3 Application of the Method to Cladonia Specimens

The applicability of the protocol to different types of lichens was tested using *Cladonia* specimens. The DNA was extracted from podetia of CkuR and Csu1 (see Table 1 for the amounts used). The experiments on *Usnea* specimens indicated that changing the lysis buffer once after an hour of incubation is adequate to increase the yield of target DNA (Table 2). Therefore, we extracted DNA from *Cladonia* specimens with the following modifications. The lysates were centrifuged at 14,000 rpm for 2 min after an hour of incubation. The supernatants were removed, and the pellets were resuspended in fresh lysis buffer. The supernatants (DNA extracts: supCkuR and supCsu1 in Table 1) were collected into 1.5 mL DNA low binding tubes after the 24 h incubation at 37°C and were subjected to DNA purification.

**TABLE 2:** Proportions of the symbiont‐derived reads in the libraries with different lysis treatments (sequenced on an iSeq 100 sequencing platform).

	UhA1		UhA5		UhA24	
	Mycobiont	Photobiont	Mycobiont	Photobiont	Mycobiont	Photobiont
Buffer exchange	0		1		2	
Total incubation (hrs)	24		5		24	
Total reads	3,180,328		647,002		426,784	
No. of reads after trim	3,180,296		647,000		426,780	
Aligned reads	109,373	472,249	51,136	105,950	37,698	79,375
Aligned reads (%)	3.44	14.85	7.90	16.38	8.83	18.60
Average length (bp)	36.96	44.47	34.49	39.07	36.26	40.27

### 2.4 DNA Purification

In the following DNA purification steps, we used 1.5 mL low binding tubes (DNA LoBind Tubes, Eppendorf) due to low DNA concentrations. The DNA extracts were centrifuged at 14,000 rpm for 2 min. The supernatants were loaded on Amicon Ultra Centrifugal Filters 4 mL/30,000 NMWL (Millipore) with 3.8 mL TE pH 8.0 (NIPPON GENE) and centrifuged at 2500 rpm until the volume of the filtrates became ≤ 100 μL. The filtrates were purified using MinElute PCR Purification Kit (QIAGEN) following the manufacturer's instructions. The DNA was eluted with 20 μL 0.1× TE buffer and stored at −20°C until use.

### 2.5 DNA Library Construction and High‐Throughput Sequencing

The DNA extracts used to construct each library are listed in Table 1. We constructed libraries using NEBNext Ultra II DNA Library Prep Kit for Illumina and NEBNext Multiplex Oligos for Illumina (96 Unique Dual Index Primer Pairs) (New England BioLabs) with the following modifications to the manufacturer's instructions. In the “Cleanup of Adaptor‐ligated DNA without Size Selection (for input ≤ 50 ng)” section, 180 μL of AMPure XP beads (Beckman Coulter) were added instead of 87 μL in the step 3B.1. Likewise, in the “Cleanup of PCR Reaction” section, 60 μL of AMPure XP beads were added instead of 45 μL in the step 5.2. We used higher amounts of AMPure XP beads to collect short DNA fragments expected from historical lichen specimens. Proportions of the symbiont‐derived reads to the total sequenced reads in libraries were estimated using an iSeq 100 sequencing platform (150 bp paired‐end reads). For resequencing, we separately constructed three DNA libraries per specimen in order to counteract the reduction of complexity in DNA fragments during the PCR amplification. The three libraries of each specimen were pooled and then purified using an equal volume of AMPure XP beads. The pooled libraries were sequenced on an Illumina Hiseq X sequencing or Novaseq X platform (150 bp paired‐end reads).

### 2.6 Alignment of Sequencing Data

Raw reads were trimmed using the default settings of CLC Genomics Workbench v23.0.2 (QIAGEN). Trimmed reads were aligned to the mycobiont and photobiont reference genomes of *U. hakonensis* (accession no. GCA_013423325 and GCA_002118135) assembled in our previous studies (Kono et al. 2017, 2020), and *C. kurokawae* assembled in this study (accession no. BAAIEH010000001–BAAIEH010000837 and BAAIEI010000001–BAAIEI010001486), by changing the length and similarity fractions to 0.9 from the default settings of CLC Genomics Workbench v23.0.2. The alignments of the three replicate libraries were merged into a single BAM file. The merged BAM files were used to calculate alignment statistics (average length of the aligned reads, fraction of the reference covered by the reads, and genome coverage) of each specimen using CLC Genomics Workbench v11.01.1 (QIAGEN).

### 2.7 Assessment of DNA Damage Patterns

We quantified nucleotide misincorporation patterns among the sequencing data of *Usnea* specimens using mapDamage v2.0 (Jónsson et al. 2013). The merged BAM files from the previous section were used as inputs with the default parameters “‐‐downsample‐seed random ‐‐length 70 ‐‐around 10 ‐‐min‐basequal 0.”

### 2.8 Phylogenetic Analysis of the Mycobionts

We downloaded Illumina short reads of the reference and closely related species from the NCBI Sequence Read Archive (SRA). The short reads of *U. hakonensis* (DRR200332), *U. cornuta* (SRR14721951), *C. kurokawae* (DRR709196), and *C. peziziformis* (SRR14721936) were aligned to the fungal reference genomes and converted to BAM files following the procedures described above. Together with the merged BAM files of the specimens, the BAM files were used in the following phylogenetic analysis. Duplicated reads in the BAM files were marked using the MarkDuplicates algorithm implemented in the GATK v4.2 (Van der Auwera and O'Connor 2020). Single‐nucleotide variant sites (SNVs) and insertions and deletions (indels) were called using the GATK HaplotypeCaller in a “‐ERC GVCF” mode that produces files in the GVCF format. The GVCF files were combined into a single file and passed to the GATK GenotypeGVCFs for joint genotyping. Genotypes were filtered using the VCFtools (Danecek et al. 2011) with parameters “‐remove‐indels ‐‐max‐missing 1 ‐‐minGQ 8 ‐‐minDP 5 ‐‐maxDP 1000”. The genotype table was converted to a Phylip (Interleaved) file using Tassel v5.0 (Bradbury et al. 2007). Phylogenetic trees were constructed using PhyML (Guindon et al. 2010) with the best model (HKY85) selected by MEGA X (Kumar et al. 2018) followed by 100 bootstrap replicates. The data we used in the phylogenetic analysis is listed in Table S1.

## 3 Results

### 3.1 Optimization of DNA Extraction Protocols

We first tested the efficacy of protocols using 4 mg of UhA. The concentration of a DNA extract (supUhA1) after the DNA purification was 0.384 ng/μL, and 6.91 ng of which was used to construct a DNA library (UhA1). Ten cycles of PCR amplification in the PCR enrichment step were adequate to obtain a DNA library of the concentration 2.19 ng/μL (Table 1). We obtained 3,180,296 reads (150 bp paired‐end) after trimming. Of the trimmed reads, 3.44% and 14.85% were aligned to the mycobiont and photobiont reference genomes, respectively (Table 2). The average lengths of the aligned reads are 36.96 and 44.47 bp for the mycobiont and the photobiont, respectively (Table 2), consistent with a previous study that reported high fragmentation of DNA in old lichen specimens (Kistenich et al. 2019).

Given the successful result, we constructed three DNA libraries in total for each of UhA and UhiL (Table 1). After trimming, we obtained 577,203,280 and 828,300,460 reads for UhA and UhiL, respectively, as a total of the three libraries (Table 3). In UhA, 2.68% and 11.82% of the reads were aligned to the mycobiont and the photobiont reference genomes, whereas the proportions are 5.11% and 7.78% in UhiL. The aligned reads of UhA and UhiL cover 73% and 81% of the mycobiont genome with average coverage 14.73× and 39.24×, respectively, while the reads cover more than 90% of the photobiont genome with average coverage over 40× (Table 3). Although DNA fragmentation is comparably intense as ancient DNA in UhA and UhiL, the frequencies of C to T/G to A substitutions at 5′/3′ ends were below 0.02 among the aligned reads, indicating the absence of typical miscoding lesions (Figure S1).

**TABLE 3:** Alignment statistics for the fungal and algal reference genomes.

	UhA		UhiL		CkuR		Csu1	
	Mycobiont	Photobiont	Mycobiont	Photobiont	Mycobiont	Photobiont	Mycobiont	Photobiont
No. of reads in total	577,215,276		828,317,204		1,285,703,014		1,053,275,480	
No. of reads after trim (paired)	577,203,280		828,300,460		1,285,470,696		1,053,080,774	
No. of aligned reads	15,456,331	68,253,919	42,305,402	64,475,607	295,541,695	38,558,799	245,465,337	54,781,855
% of aligned reads	2.68	11.82	5.11	7.78	22.99	3.00	23.31	5.20
Average read length (bp)	40.60	45.17	39.46	43.57	46.16	45.9	43.53	41.81
Fraction of reference covered (%)	73	92	81	94	99	99	97	98
Average coverage	14.73	49.67	39.24	40.23	424.67	31.45	332.33	40.58

To maximize the yield of the reads derived from the targets (mycobionts and photobionts), we changed the lysis buffer that contains non‐target DNA twice during the lysis step (Figure 2). The concentration of DNA in a DNA extract (supUhA5) after the first buffer exchange was 3.18 ng/μL, whereas the concentration was 0.644 ng/μL in an extract (supUhA24) after the second buffer exchange (Table 1). The iSeq 100 sequencing of UhA5 and UhA24 yielded 647,000 and 426,780 reads after trimming, respectively, of which 7.90% and 8.83% were aligned to the mycobiont genome, while 16.38% and 18.60% were aligned to the photobiont genome (Table 2).

### 3.2 Phylogenetic Positions of the *Usnea* Specimens

**FIGURE 3:**
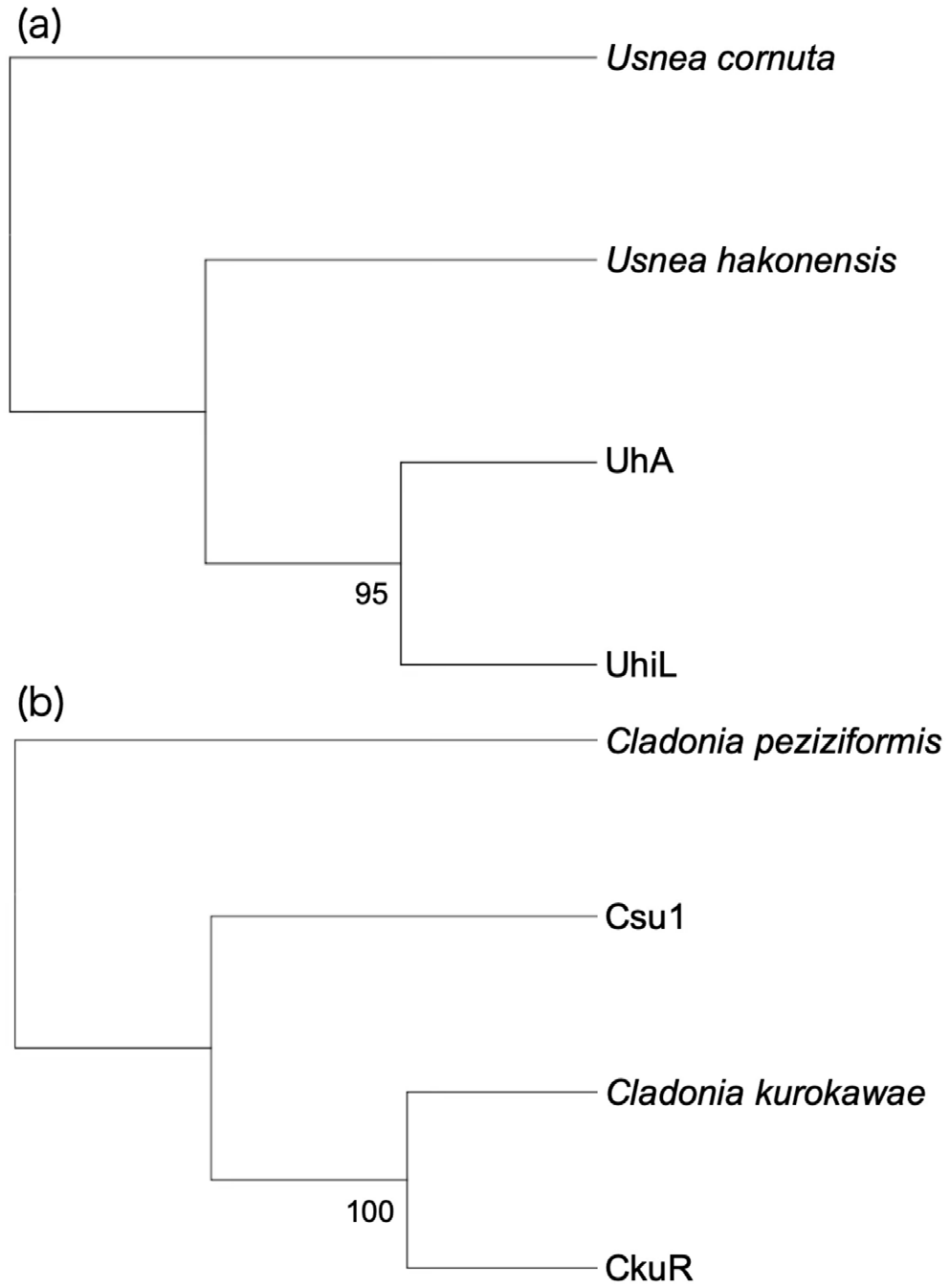
Phylogenetic positions of the specimens sequenced in this study. The ML trees of (a) *Usnea* and (b) *Cladonia* were constructed using 792,245 and 410,705 SNVs, respectively. Node values indicate bootstrap support.

To confirm phylogenetic positions of the genomic data retrieved from the specimens, we constructed a fungal tree using SNVs extracted from the short‐reads aligned to the reference genome of *U. hakonensis*. In total, 792,245 sites were used to construct a tree in which both of the specimens cluster together with a 95% bootstrap support, presenting close relations to the reference *U. hakonensis* (Figure 3).

### 3.3 Application of the Method to *Cladonia* Specimens

We determined the mycobiont and the photobiont genomes of *Cladonia kurokawae* to be used as the references. De novo assembly of 69.5 M and 69.3 M 150 bp paired‐end reads presented mycobiont and photobiont genomes of size 32.0 and 55.6 Mb, N50 as 143.9 and 98.3 kb, with average coverage 318× and 180×, respectively. Completeness of the fungal and algal genomes indicated by Benchmarking Universal Single‐Copy Orthologs (BUSCO) assessment was 97.4% (Ascomycota set) and 85.4% (Chlorophyta set), respectively.

For resequencing, we obtained 1,285,470,696 and 1,053,080,774 trimmed reads for CkuR and Csu1, respectively (Table 3). In CkuR, 22.99% and 3.00% of the reads were aligned to the mycobiont and the photobiont reference genomes, whereas the proportions are 23.31% and 5.20% in Csu1. The aligned reads of CkuR and Csu1 cover 99% and 97% of the mycobiont genome with average coverage 424.67× and 332.33×, respectively, while the reads cover 99% and 98% of the photobiont genome with average coverage 31.45× and 40.58×, respectively (Table 3). A fungal tree using 410,705 SNVs shows that the independent culture of *C. kurokawae* we sequenced in this study to assemble the reference genome clusters together with the holotype specimens (CkuR) with a 100% bootstrap support (Figure 3).

## 4 Discussion

High DNA fragmentation of historical lichen specimens has long hindered the molecular investigation of museum collections in lichenology. Only a few studies have successfully amplified and sequenced limited DNA regions from specimens older than 50 years (Bendiksby et al. 2014; Kistenich et al. 2019; Cubero et al. 1999; Sohrabi et al. 2010). To the best of our knowledge, no successful whole genome sequencing of historical lichen specimens has been reported. Here, considering the potential benefits of whole genomic information for future lichen studies, we attempted to establish an experimental method to perform the whole genome sequencing of historical lichen specimens. Our method required as little as 4 mg of a specimen to construct one DNA library and has proven effective for specimens of different species and ages, including types. Although proportions of the mycobiont and photobiont‐derived reads within the total reads ranged from 2.68% to 23.31% and 3.00% to 11.82%, respectively, at least 73% of the fungal and 92% of the algal reference genomes were covered by reads, providing enough sites to perform phylogenetic analysis at the genomic level. The first simultaneous investigation of fungal and algal DNA in historical lichen specimens revealed that the fragmentation of DNA could proceed differently between the two symbionts and among lichen species. In the two *U. hakonensis* specimens, the average length of the reads aligned to the reference genomes is slightly longer in the photobiont, and the proportions of the reads aligned to the algal genome are higher than those aligned to the fungal genome (Table 3). In lichens, the mycobiont constitutes a large proportion of the total biomass, whereas the photobiont is estimated to constitute as little as 10% of it (Green et al. 2008). Their difference in the biomass is also reflected in the proportions of reads in metagenomic data obtained from freshly collected lichen samples (Tagirdzhanova et al. 2021). Taking this into account, our results for the two *U. hakonensis* samples show that the algal DNA is well preserved compared to the fungal DNA. Meanwhile, in the two *Cladonia* specimens, such a reversed trend of biomass and read numbers was not observed. Even if we account for differences in the DNA extraction protocol, we obtained much higher proportions of the mycobiont‐derived reads in the *Cladonia* samples than in the *Usnea* samples. We assume this was caused by apothecia, which were only present in the *Cladonia* specimens (Figure 1). Fragmented DNA extracted from ancient remains often presents high frequencies of cytosine to thymine substitutions at 5′ ends and complementary guanine to adenine substitutions at 3′ ends, as a result of hydrolytic deamination of cytosine residues to uracils, which occurs on single‐stranded overhanging ends of DNA fragments (Dabney et al. 2013). This typical damage pattern in ancient DNA, which proceeds with age, is however not detected in the *Usnea* specimens possibly due to their relatively young age.

The short average length of the reads aligned to the mycobiont and photobiont genomes (39.46–46.16 bp) in the present study is consistent with high fragmentation of DNA in old lichen specimens reported by Kistenich et al. (2019). Such short fragments of DNA are unsuitable for PCR‐based methods that amplify long sequences between primers for specific marker genes, giving an advantage to our method that uses whole genome sequencing technique to sequence fragmented DNA itself and obtain genome‐wide sequencing data. Another advantage of our method is that we only run 11 PCR cycles at maximum to enrich adaptor‐ligated DNA during the library construction. PCR‐based methods that often run more than 30 cycles to amplify target DNA sequences have an inherent risk of amplifying sequences of non‐target organisms that have attached to specimens before and after the collection.

Potential constraints of our method are the limited availability of specimens and higher cost of sequencing compared to PCR‐based methods. The amount of material that can be used for destructive DNA extraction is often restricted for valuable specimens such as holotypes, while DNA library construction with reduced PCR cycles is expected to be more successful using a larger amount of input DNA. Moreover, even with a successful DNA library, the proportion of symbiont‐derived reads within total sequenced reads is tends to be low. In the present study, a maximum of 23.31% and 11.82% of the total reads were aligned to the mycobiont and the photobiont genomes, respectively (Table 3). To achieve sufficient genome coverage for the target species, we sequenced approximately 100 G bite data for each specimen. This is 25 times more than sequencing a single mycobiont genome at 100× coverage. Additionally, since our method relies on the accurate alignment of short reads to reference genomes, sequencing costs may increase further if reference genomes are not already available in the public databases. Our optimized protocols have succeeded in increasing the proportion of mycobiont‐derived reads from 3.44% in UhA1 to 8.83% in UhA24 (Table 2) by eliminating DNA from non‐target organisms during lysis steps. A relatively mild increase in the proportion of mycobiont‐derived reads between UhA5 and UhA24 revealed that the buffer exchange after one hour of incubation is most effective in removing DNA from non‐target organisms. Given these results, we recommend optimizing DNA extraction protocols to reduce the cost of sequencing.

Although the general utility of this method should be tested with historical lichen specimens of diverse taxa and ages, we believe it could become a breakthrough in the utilization of millions of lichen specimens stored in herbaria all over the world.

## Author Contributions

**Mieko Kono:** conceptualization (lead), data curation (lead), formal analysis (lead), funding acquisition (equal), investigation (lead), methodology (lead), project administration (equal), validation (lead), visualization (lead), writing – original draft (lead). **Yoshihito Ohmura:** conceptualization (lead), funding acquisition (equal), project administration (equal), resources (lead), visualization (supporting), writing – review and editing (equal). **Yohey Terai:** conceptualization (lead), data curation (lead), formal analysis (lead), funding acquisition (equal), investigation (lead), methodology (lead), project administration (equal), validation (lead), visualization (supporting), writing – original draft (supporting).

## Conflicts of Interest

The authors declare no conflicts of interest.

## Supporting information


**Figure S1:** Frequencies of C to T/G to A mutations at 5′/3′ ends of the reads aligned to the reference genomes. The xand y‐ axis show the relative position of the reads and the mutation frequencies at each position, respectively. (a) UhA reads, mycobiont genome, (b) UhA reads, photobiont genome, (c) UhiL reads, mycobiont genome, (d) UhiL reads, photobiont genome.

**Table S1:** Genomic sequences and Illumina short reads used in the phylogenetic analysis of the myconbionts.

## Data Availability

All raw reads sequenced in this study are deposited in the DDBJ Sequence Read Archive under BioProject PRJDB19959 and PRJDB20668. Benefits generated: Benefits from this research accrue from the sharing of our data on public databases as described above.
