# Supplementary material for: The First Whole Genome Sequencing of Historical Lichen Specimens Enables Genome‐Wide Analysis of Fungal and Algal Symbionts
Source: Ecol Evol. 2025 Sep 22;15(9):e72216. doi: 10.1002/ece3.72216 (PMC12453609; doi:10.1002/ece3.72216)
Supplement: Supplementary file 1 — Figure S1: Frequencies of C to T/G to A mutations at 5′/3′ ends of the reads aligned to the reference genomes. The xand y‐ axis show the relative position of the reads and the mutation frequencies at each position, respectively. (a) UhA reads, mycobiont genome, (b) UhA reads, photobiont genome, (c) UhiL reads, mycobiont genome, (d) UhiL reads, photobiont genome. [file ECE3-15-e72216-s002.pdf]

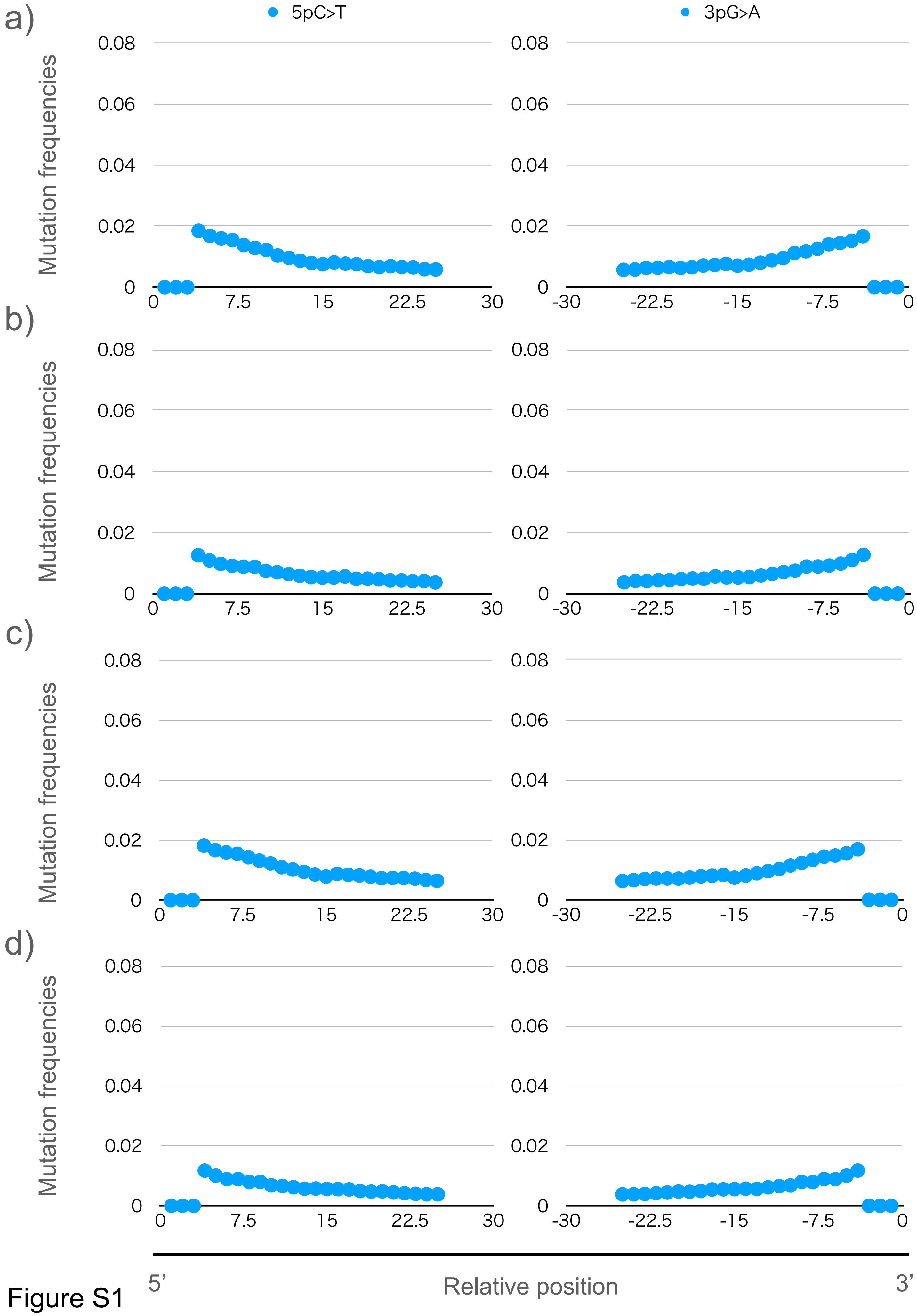

Figure S1

Frequencies of C to T/G to A mutations at 5'/3' ends of the reads aligned to the reference genomes. The x- and y- axis show the relative position of the reads and the mutation frequencies at each position, respectively. (a) UhA reads, mycobiont genome, (b) UhA reads, photobiont genome, (c) UhiL reads, mycobiont genome, (d) UhiL reads, photobiont genome.
